# Basic MAC Scheme for RF Energy Harvesting Wireless Sensor Networks: Throughput Analysis and Optimization†

**DOI:** 10.3390/s19081822

**Published:** 2019-04-16

**Authors:** Cheon Won Choi

**Affiliations:** Department of Applied Computer Engineering, Dankook University, Yongin 31116, Korea; cchoi@dku.edu; Tel.: +82-31-8005-3659

**Keywords:** wireless sensor network, RF energy harvesting, MAC scheme, ALOHA, renewal theory, throughput, optimal back-off time, effectiveness, shaping

## Abstract

Traditionally, how to reduce energy consumption has been an issue of utmost importance in wireless sensor networks. Recently, radio frequency (RF) energy harvesting technologies, which scavenge the ambient RF waves, provided us with a new paradigm for such networks. Without replacement or recharge of batteries, an RF energy harvesting wireless sensor network may live an eternal life. Against theoretical expectations, however, energy is scarce in practice and, consequently, structural naiveté has to be within a MAC scheme that supports a sensor node to deliver its data to a sink node. Our practical choice for the MAC scheme is a basic one, rooted in ALOHA, in which a sensor node simply repeats harvesting energy, backing off for a while and transmitting a packet. The basic medium access control (MAC) scheme is not able to perfectly prevent a collision of packets, which in turn deteriorates the throughput. Thus, we derive an exact expression of the throughput that the basic MAC scheme can attain. In various case studies, we then look for a way to enhance the throughput. Using the throughput formula, we reveal that an optimal back-off time, which maximizes the total throughput, is not characterized by the distribution but only by the mean value when the harvest times are deterministic. Also, we confirm that taking proper back-off times is able to improve the throughput even when the harvest times are random. Furthermore, we show that shaping the back-off time so that its variance is increased while its mean remains unchanged can help ameliorate the throughput that the basic MAC scheme is able to achieve.

## 1. Introduction

A wireless sensor network consists of sink nodes and sensor nodes [1]. In the network, a sensor node gathers information in the vicinity and delivers it to a sink node. To carry out such a mission, a sensor node is typically equipped with a battery that powers the sensor node itself. A wireless sensor network is often deployed in a harsh environment, where batteries are hardly recharged or replaced. Consequently, issues on energy consumption have arisen in designing a wireless sensor network. Then, many efforts have been made to solve the energy problems in a wireless sensor network, most of which were focused on devising efficient communication schemes to reduce energy consumption and ultimately to extend the lifetime of the network [2,3,4,5].

Recently, radio frequency (RF) energy harvesting technologies provided us with a new paradigm for wireless sensor networks [6,7,8]. These technologies are mainly divided into two categories: a category of technologies which enable sensor nodes to harvest energy by scavenging ambient RF waves and the other category of technologies which designate external RF sources to supply energy to sensor nodes by radiating RF waves. In [9], the capacitor leakage problem was taken into account when sensor nodes harvest ambient RF energy. In [10,11], it was assumed that a sensor node is able to extract information and harvest energy as well from the signal transmitted by another node. In [12], cooperation of licensed and unlicensed users was investigated in a cognitive network where unlicensed users harvest ambient energy to charge their batteries. In [13], a cognitive network was considered in which a sensor node senses the spectrum and harvests energy in the spectral band used by access points. By harvesting RF energy, a wireless sensor network can overcome the battery constraint and is, at least theoretically, able to live eternally. However, harvested energy is not enough to directly power a sensor node but merely capable of charging an internal capacitor, especially, when a sensor node harvests energy by scavenging ambient RF waves. Moreover, it takes quite a long time to charge the capacitor compared to the time for which the sensor node actually participates in communicating with a sink node [14,15]. As a result, energy is still scarce in practice and a sensor node should repeat the transition between the long harvesting state and the short communicating state. For example, it was reported that the harvesting state has to last roughly 20 times longer than the communicating state in [16].

A medium access control (MAC) scheme is needed for a sensor node to deliver information to a sink node, especially, in a wireless sensor network where several sensor nodes attempt to send their data to a common sink node. In an RF energy harvesting wireless sensor network, which suffers from a scarcity of energy, signaling is not easily provided between sink and sensor nodes. Furthermore, a sensor node is hardly able to exchange information with another sensor node. Thus, a sophisticated MAC scheme of scheduling-type is not suitable for supporting a sensor node to deliver data to a sink node. Structural simplicity has to be within the MAC scheme in an RF energy harvesting wireless sensor network. In the literature, a set of MAC schemes have been reported for energy harvesting wireless sensor networks [17,18,19,20,21,22,23,24,25,26,27,28,29,30,31,32]. In [17], a polling-based MAC scheme was introduced in which the sink node gives a chance of transmitting data with a certain probability to a sensor node. Then, the proposed scheme was evaluated under the assumption that sensor nodes harvest solar or thermal energy. As MAC schemes for energy harvesting wireless sensor networks, time division multiple access (TDMA) and framed and slotted ALOHA were considered in [18]. To maintain slotted time structure, such schemes were also complemented by periodic signaling of the sink node destined to sensor nodes. In [19,29], MAC schemes based on carrier sense multiple access with collision avoidance (CSMA/CA) were proposed. These schemes were designed to assign an external RF source to supply RF energy to a sensor node. In [20], a MAC scheme based on CSMA/CA was addressed. To behave according to the scheme, a sensor node was assumed to continuously harvest energy from surrounding environments. In [21,26], TDMA and slotted CSMA/CA were respectively used for sensor nodes to send their data to the sink node, where the sink node was assumed to be able to receive data from sensor nodes and send RF energy to sensor nodes simultaneously. In [22], a MAC scheme using two different polling methods was suggested for supporting two priority classes in a wireless body area network. In this work, sensor nodes were assumed to harvest energy in human body. Also, a simulation method was employed to evaluate the suggested MAC scheme. In [23], relays were deployed at a wireless body area network. Then, a MAC scheme was proposed for cooperative communications. In this work, relays were assumed to harvest thermal or biochemical energy in human body. Also, the proposed MAC scheme was evaluated by use of a simulation method. In [27], duty cycles were investigated in energy harvesting wireless sensor networks where sensor nodes harvest photovoltaic energy. In [28], a MAC scheme which is compatible with IEEE 802.15.4 standard was proposed for RF energy harvesting wireless sensor networks. To use the scheme, the authors assumed that an external RF source supply energy to sensor nodes by radiating RF waves. In [30], a MAC scheme based on distributed coordination function in IEEE 802.11 was proposed for sensor nodes to send data to an access point. In this MAC scheme, access points were assumed to supply RF energy to sensor nodes. In [31], a MAC scheme was introduced to meet the quality-of-service (QoS) requirement per priority class. The scheme was then evaluated under the assumption that sensor nodes harvest solar energy. In [32], a MAC scheme based on slotted ALOHA was reported for energy harvesting wireless sensor network, where the sink node was assumed to be able to receive data from sensor nodes and send RF energy to sensor nodes simultaneously. Considerable effort has been made to device MAC schemes for wireless sensor networks in which sensor nodes harvest solar energy or receive RF energy from external RF sources. On the other hand, a limited number of MAC schemes have been reported for wireless sensor networks where sensor nodes harvest RF energy only by scavenging ambient RF waves. Some of the MAC schemes were designed to let sensor nodes to often make a transit between transmitter and receiver modes. Unfortunately, such schemes may be rather complicated to follow in case sensor nodes can only harvest weak RF energy, e.g., −20 dBm [15], from ambient RF waves so that sensor nodes are not able to operate with being directly powered by harvested energy but also need long time, e.g., 3 h [15], to charge its capacitor. Some other MAC schemes were devised based on the technologies which enable a sink node to receive data from sensor nodes and send RF energy to sensor nodes simultaneously. These technologies have not yet fully matured to be hired in various wireless environments [33,34]. Also, they are not applicable to a wireless sensor network in which sensor nodes scavenge ambient RF waves to harvest energy. In previous studies, simulation methods were often used to evaluate proposed MAC schemes. Compared with deriving mathematical expressions for performance measures, simulation methods are advantageous in the sense that they are able to embrace complex practical models for energy harvesting wireless sensor networks. On the other hand, deriving exact, or alternatively approximate, expressions provides a handy tool for optimization, which typically requires vast simulation results. In wireless sensor networks where sensor nodes harvest energy by scavenging ambient RF waves; However, there have been reported few results about exact expressions of performance measures, e.g., throughput, for evaluating as well as optimizing a MAC scheme.

In this paper, we consider an RF energy harvesting wireless sensor network in which sensor nodes harvest RF energy by scavenging ambient RF waves. Upon perception of such difficulties in an RF energy harvesting wireless sensor network, our practical choice for the MAC scheme is a basic contending-type MAC scheme based on pure ALOHA [35]. In the basic MAC scheme, a sensor node harvests energy from ambient RF waves, charges a capacitor, senses the environment, generates a packet, takes a back-off time, and transmits the packet. Then, the sensor node simply repeats the procedures above. (Please note that the basic MAC scheme is not identical to pure ALOHA but only employs some features of pure ALOHA including back-off times.) If two or more sensor nodes may simultaneously send their packets to a sink node, a collision of the packets takes place and the sink node may not able to identify any packet. However, the basic MAC scheme is not able to perfectly prevent such a collision of packets since it belongs to ALOHA clan. As a result, the throughput that the basic MAC scheme can attain is deteriorated by a collision of packets. Using the renewal theory, we thus derive an exact expression of the throughput that the basic MAC scheme can achieve. In various case studies, we then look for a way to enhance the throughput by use of the throughput formula. Specifically, we seek answers to the fundamental questions; what is an optimal distribution for the back-off time which maximizes the total throughput?”, “Is it possible to effectively increase the total throughput by taking back-off times?” and “how to shape the distribution for the back-off time as to improve the total throughput?” There have been classical studies on back-off methods. In [36], several back-off methods, which adjust the back-off times as retransmissions go on, were considered in networks governed by slotted ALOHA. Then, these methods are evaluated comparatively. In [37], an exponential back-off method was suggested for slotted ALOHA systems. Then, a simulation method was used to evaluate the proposed back-off method. In [38], back-off methods, which dynamically control the back-off time according to the result of packet delivery, were investigated in slotted ALOHA networks. In [39], a back-off method, which can be adopted at CSMA/CA was proposed for IEEE 802.15.4 networks. In [40], an adaptive back-off method was explored to be used, in IEEE 802.11 wireless local area networks. In the basic MAC scheme, a sensor node is not acknowledged about its attempt to deliver a packet to the sink node. However, most of the previous studies have focused on adaptive back-off methods that rely on the result of packet delivery. The basic MAC scheme is based on pure ALOHA which works on a continuous time structure. On the other hand, many previous works have assumed MAC schemes operating on discrete time structures, e.g., slotted ALOHA. As far as we know, there has been no intensive study on back-off methods which will be employed in a contending-type MAC scheme, rooted in pure ALOHA, for RF energy harvesting wireless sensor networks.

In Section 2, we describe a basic MAC scheme for supporting sensor nodes to deliver their data to a sink node in a wireless sensor network where sensor nodes harvest RF energy by scavenging ambient RF waves. In Section 3, using the renewal theory, we derive an exact expression of the throughput that the basic MAC scheme is able to achieve. Also, we exactly present the throughput in a closed form when harvest times, back-off times and transmission times are exponentially distributed. Section 4 is devoted to case studies for seeking strategies to improve the throughput by controlling back-off times. First, we find an optimal distribution for the back-off time when harvest times are deterministic. Secondly, we examine the impact of taking back-off times on the throughput when harvest times are random. Thirdly, we investigate the effect of variance of back-off time on the throughput when the sum of harvest and back-off times are governed by an Erlang distribution.

## 2. Basic MAC Scheme

**Figure 1:**
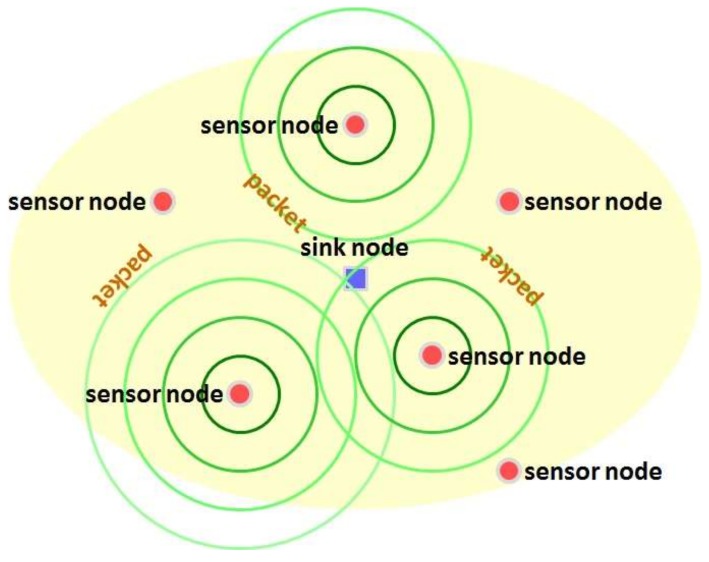
Configuration of RF energy harvesting wireless sensor network.

**Figure 2:**
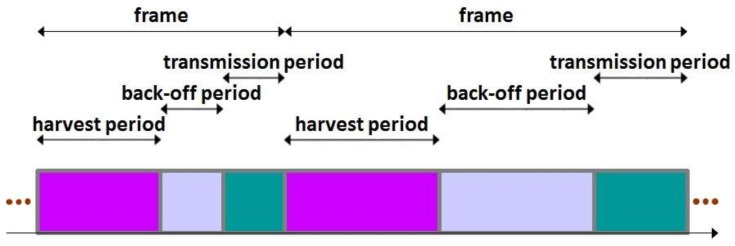
Time structure adopted by basic MAC scheme.

**Figure 3:**
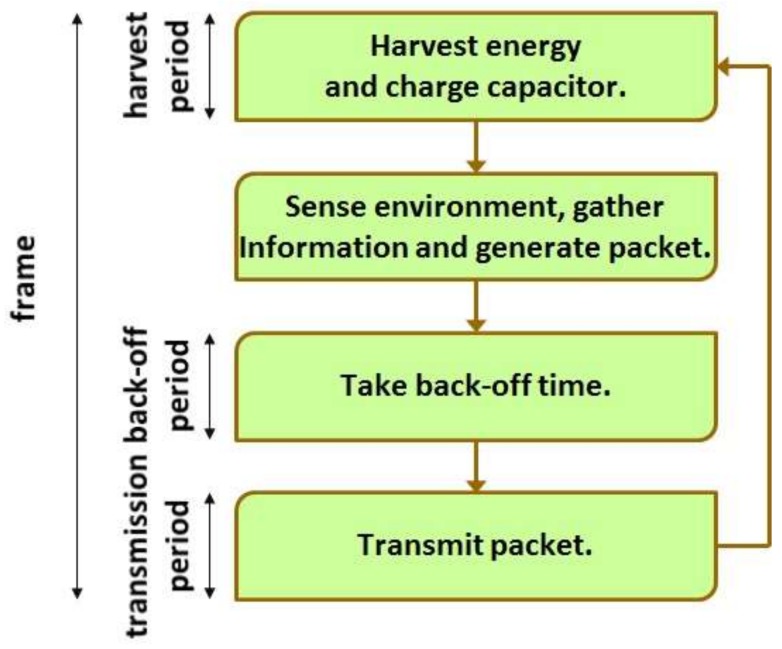
Behavior of basic MAC scheme.

Consider an RF energy harvesting wireless sensor network which consists of a single sink node and many sensor nodes. The sink node is laid at the center of the network and sensor nodes are scattered around the sink node. An exemplary configuration of the RF energy harvesting wireless sensor network is illustrated in Figure 1. In the network, a sensor node harvests energy by scavenging ambient RF waves. By consuming the harvested energy, the sensor node gathers information in its vicinity and then sends the collected data to the sink node. For such delivery of data, we consider a contending-type MAC scheme, rooted in ALOHA, as follows:

At each sensor node, time is divided into frames and a frame is partitioned into harvest period, back-off period and transmission period. (See Figure 2.) When a frame starts at a sensor node, a harvest period of the sensor node also begins. During the harvest period, the sensor node harvests RF energy and charges the internal capacitor. Please note that the harvest period lasts until the sensor node accumulates as much energy as it can transmit a packet. As the harvest period ends, the sensor node senses the environment, collects information and encapsulates the information into a packet. Then, a back-off period of the sensor node starts. The sensor node intentionally waits for the back-off period in the expectation for reducing the possibility of packet collision. When the back-off period is over, a transmission period of the sensor node begins and the sensor node finally transmits the packet to the sink node. As the transmission period ends, the next frame starts. Figure 3 summarizes the behavior of the basic MAC scheme considered in the paper.

According to the basic MAC scheme, two or more transmission periods of different sensor nodes may overlap. Then, the packets which are sent during these transmission periods collide and the sink node may not be able to identify any packet. Please note that the sink node returns no acknowledgement message even if it receives and identifies a packet. Thus, as a sensor node finishes transmitting a packet, the sensor node immediately discards the packet and never retransmits it. Consequently, a sensor node may lose a packet if the packet is involved in a collision.

## 3. Throughput Analysis

In the basic MAC scheme, each sensor node independently transmits a packet as stated in Section 2. Inevitably, a packet may collide with others and the sink node may identify none of the packets involved in the collision. Moreover, a collision of packets ultimately leads to a loss of them since a sensor node never retransmits a packet. In this section, reflecting the effect of packet collision on the throughput, we derive an exact expression of the throughput that the basic MAC scheme is able to attain. Then, we present an expression of the throughput in a closed form when harvest times, back-off times and transmission times are exponentially distributed.

### 3.1. Exact Expression of Throughput

**Figure 4:**
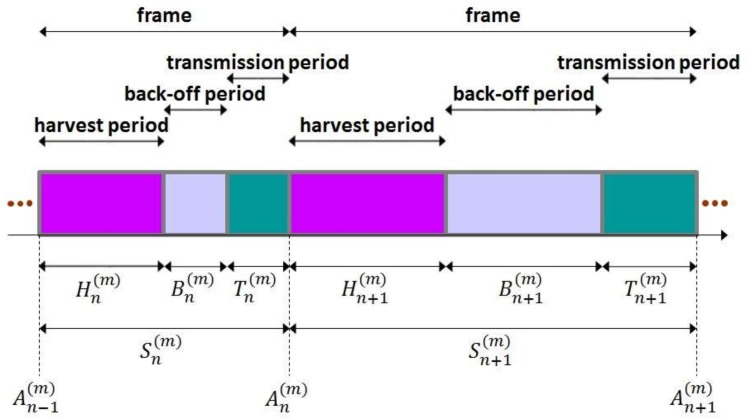
Harvest times, back-off times and transmission times.

Consider a wireless sensor network which consists of a single sink node and $M\in \left\{2,3,\cdots \right\}$ sensor nodes, denoted by $s_{1}$, ⋯, $s_{M}$. At sensor node $s_{m}$, time is divided into frames and a frame is again partitioned into harvest period, back-off period and transmission period as stated in Section 2. For k∈ℕ, let $H_{k}^{\left(m\right)}$, $B_{k}^{\left(m\right)}$ and $T_{k}^{\left(m\right)}$, respectively, denote the harvest time, back-off time and transmission time (i.e., the lengths of harvest period, back-off period and transmission period) in the kth frame at sensor node $s_{m}$. Set

(1) $$S_{k}^{\left(m\right)}≜H_{k}^{\left(m\right)}+B_{k}^{\left(m\right)}+T_{k}^{\left(m\right)}$$

for k∈ℕ. Assume that the time for sensing the environment, gathering information and generating a packet is negligible. Then, $S_{k}^{\left(m\right)}$ represents the length of the kth frame at sensor node $s_{m}$. Also, set $A_{0}^{\left(m\right)}=0$ almost surely and define

(2) $$A_{n}^{\left(m\right)}≜\overset{n}{\underset{k=1}{\sum }}S_{k}^{\left(m\right)}$$

for n∈ℕ. Then, $A_{n}^{\left(m\right)}$ indicates the time that the nth frame starts. Please note that $\left\{A_{n}^{\left(m\right)},n=0,1,\cdots \right\}$ forms a point process [41]. Figure 4 illustrates harvest times, back-off times and transmission times.

Suppose that a sensor node needs a fixed amount of energy, denoted by ε, to transmit a packet. Recall that a sensor node keeps harvesting energy until it accumulates a necessary amount of energy for transmitting a packet. Let $J_{t}^{\left(m\right)}$ represent the amount of energy that sensor node $s_{m}$ is able to harvest in $\left(0,t\right]$ for $t\in \left[0,\infty \right)$. Then, the harvest time $H_{k}^{\left(m\right)}$ can be expressed by

(3) $$H_{k}^{\left(m\right)}=\min{\{}_{}^{}u\in \left(0,\infty \right):J_{A_{k}^{\left(m\right)}+u}^{\left(m\right)}-J_{A_{k}^{\left(m\right)}}^{\left(m\right)}\geq \varepsilon \}$$

for k∈ℕ. To characterize the harvest times, we need to model the amount of harvested energy $J_{t}^{\left(m\right)}$. In the literature, some models have been introduced on the amount of harvested energy [42]. Since we aim to derive an exact expression of the throughput, we employ a tractable model of ${\{}_{}^{}J_{t}^{\left(m\right)},t\geq 0\}$ as follows. Set $J_{0}^{\left(m\right)}=0$ almost surely and assume that ${\{}_{}^{}J_{t}^{\left(m\right)},t\geq 0\}$ is a non-decreasing Lévy process, i.e., a subordinator [43]. Then, the harvest times $H_{1}^{\left(m\right)}$, $H_{2}^{\left(m\right)}$, ⋯ are mutually independent and identically distributed. Let $H^{\left(m\right)}$ denote a random variable such that $H^{\left(m\right)}=H_{k}^{\left(m\right)}$ in distribution. Let $F_{H}^{\left(m\right)}$ denote the distribution function of $H^{\left(m\right)}$, i.e.,

(4) $$F_{H}^{\left(m\right)}\left(x\right)=P{(}_{}^{}H^{\left(m\right)}\leq x)$$

for x∈ℝ. At sensor node $s_{m}$, suppose that the back-off times $B_{1}^{\left(m\right)}$, $B_{2}^{\left(m\right)}$, ⋯ are mutually independent and identically distributed. Let $B^{\left(m\right)}$ denote a random variable such that $B^{\left(m\right)}=B_{k}^{\left(m\right)}$ in distribution and $F_{B}^{\left(m\right)}$ be the distribution function of $B^{\left(m\right)}$, i.e.,

(5) $$F_{B}^{\left(m\right)}\left(x\right)=P{(}_{}^{}B^{\left(m\right)}\leq x)$$

for x∈ℝ. In addition, assume that the transmission times $T_{1}^{\left(m\right)}$, $T_{2}^{\left(m\right)}$, ⋯ are mutually independent and identically distributed as a random variable $T^{\left(m\right)}$. Let $F_{T}^{\left(m\right)}$ denote the distribution function of $T^{\left(m\right)}$, i.e.,

(6) $$F_{T}^{\left(m\right)}\left(x\right)=P{(}_{}^{}T^{\left(m\right)}\leq x)$$

for x∈ℝ. Under the assumption that ${\{}_{}^{}H_{k}^{\left(m\right)},k=1,2,\cdots \}$, ${\{}_{}^{}B_{k}^{\left(m\right)},k=1,2,\cdots \}$ and ${\{}_{}^{}T_{k}^{\left(m\right)},k=1,2,\cdots \}$ are independent and identically distributed sequences, the lengths of frames $S_{1}^{\left(m\right)}$, $S_{2}^{\left(m\right)}$, ⋯ are also mutually independent and identically distributed. Thus, ${\{}_{}^{}A_{n}^{\left(m\right)},n=0,1,\cdots \}$ becomes a renewal point process [41]. Let $S^{\left(m\right)}$ denote a random variable such that $S^{\left(m\right)}=S_{k}^{\left(m\right)}$ in distribution for all k∈ℕ. Also, let $F_{S}^{\left(m\right)}$ represent the distribution function of $S^{\left(m\right)}$, i.e.,

(7) $$F_{S}^{\left(m\right)}\left(x\right)=P{(}_{}^{}S^{\left(m\right)}\leq x)$$

for x∈ℝ.

For $t\in \left(0,\infty \right)$, define a random variable $Q_{t}^{\left(m\right)}$ as follows:

(8) $$Q_{t}^{\left(m\right)}=\min{\{}_{}^{}n\in \mathbb{N}:A_{n}^{\left(m\right)}>t\}.$$

Also, let $V_{t}^{\left(m\right)}$ denote the time elapsed from t until the next frame starts. Then, $V_{t}^{\left(m\right)}$, which is called the forward recurrence time [44] associated with the renewal point process ${\{}_{}^{}A_{n}^{\left(m\right)},n=0,1,\cdots \}$, can be expressed by

(9) $$V_{t}^{\left(m\right)}=A_{Q_{t}^{\left(m\right)}}^{\left(m\right)}-t$$

for $t\in \left(0,\infty \right)$. Recall that $T_{k}^{\left(m\right)}$ is the time needed by sensor node $s_{m}$ to transmit a packet in the kth frame. Define

(10) $$W_{t}^{\left(m\right)}≜\left\{\begin{array}{cc} V_{t}^{\left(m\right)}-T_{Q_{t}^{\left(m\right)}}^{\left(m\right)} & \mathrm{if}\;V_{t}^{\left(m\right)}>T_{Q_{t}^{\left(m\right)}}^{\left(m\right)}\\ 0 & \mathrm{otherwise}\; \end{array}\right.$$

for $t\in \left(0,\infty \right)$. Then, $W_{t}^{\left(m\right)}$ represents the time elapsed from t until sensor node $s_{m}$ begins to transmit a packet in the current frame. (If sensor node $s_{m}$ already started transmitting a packet before t, $W_{t}^{\left(m\right)}$ is set to be 0 almost surely.) Apparently, ${\{}_{}^{}W_{t}^{\left(m\right)},t\geq 0\}$ is a regenerative process [44] associated with the renewal point process ${\{}_{}^{}A_{n}^{\left(m\right)},n=0,1,\cdots \}$. Thus, $W_{t}^{\left(m\right)}$ satisfies the renewal equation [44] as follows.

(11) $$P{(}_{}^{}W_{t}^{\left(m\right)}>y)=\theta^{\left(m\right)}\left(t\right)+\overset{t}{\underset{0}{\int }}P{(}_{}^{}W_{t-s}^{\left(m\right)}>y)dF_{S}^{\left(m\right)}\left(s\right)$$

for $t\in \left(0,\infty \right)$, where

(12) $$\theta^{\left(m\right)}\left(t\right)=P{(}_{}^{}W_{t}^{\left(m\right)}>y,A_{1}^{\left(m\right)}>t)$$

for $t\in \left(0,\infty \right)$. Since $\theta^{\left(m\right)}\left(t\right)$ is bounded, the renewal equation in (11) has a unique solution. For all $t\in \left(0,\infty \right)$, set $R_{1}^{\left(m\right)}\left(t\right)=F_{S}^{\left(m\right)}\left(t\right)$ and define

(13) $$R_{k+1}^{\left(m\right)}\left(t\right)=\overset{t}{\underset{0}{\int }}R_{k}^{\left(m\right)}\left(t-s\right)dF_{S}^{\left(m\right)}\left(s\right)$$

for k∈ℕ. Then, the renewal function [44], denoted by $R^{\left(m\right)}$, is defined by

(14) $$R^{\left(m\right)}\left(t\right)=\overset{\infty }{\underset{k=1}{\sum }}R_{k}^{\left(m\right)}\left(t\right)$$

for $t\in \left(0,\infty \right)$. Using the renewal function in (14), the unique solution of the renewal equation is expressed by

(15) $$P{(}_{}^{}W_{t}^{\left(m\right)}>y)=\theta^{\left(m\right)}\left(t\right)+\overset{t}{\underset{0}{\int }}\theta^{\left(m\right)}\left(t-s\right)dR^{\left(m\right)}\left(s\right)$$

for $t\in \left(0,\infty \right)$. Suppose that $S^{\left(m\right)}$ is not arithmetic [44]. Please note that

(16) $$\theta^{\left(m\right)}\left(t\right)=P{(}_{}^{}H_{1}^{\left(m\right)}+B_{1}^{\left(m\right)}>t+y)=P{(}_{}^{}H^{\left(m\right)}+B^{\left(m\right)}>t+y)$$

for $t\in \left(0,\infty \right)$ and $y\in \left(0,\infty \right)$. Since harvest time $H^{\left(m\right)}$ and back-off time $B^{\left(m\right)}$ are properly distributed, $\theta^{\left(m\right)}$ is a non-negative non-increasing function of $t\in \left(0,\infty \right)$. In addition, $\overset{\infty }{\underset{0}{\int }}\theta^{\left(m\right)}\left(t\right)\;dt<\infty$. Thus, from the key renewal theorem [44], we have

(17) $$P{(}_{}^{}W_{t}^{\left(m\right)}>y)\to \frac{1}{E\left(S^{\left(m\right)}\right)}\overset{\infty }{\underset{0}{\int }}\theta^{\left(m\right)}\left(t\right)dt$$

as t→∞. Please note that

(18) $$\frac{1}{E\left(S^{\left(m\right)}\right)}\overset{\infty }{\underset{0}{\int }}\theta^{\left(m\right)}\left(t\right)dt=\frac{1}{E\left(S^{\left(m\right)}\right)}\overset{\infty }{\underset{0}{\int }}1-F_{H}^{\left(m\right)}*F_{G}^{\left(m\right)}\left(t+y\right)dt$$

from (16), where

(19) $$F_{H}^{\left(m\right)}*F_{G}^{\left(m\right)}\left(x\right)=\overset{x}{\underset{0}{\int }}F_{H}^{\left(m\right)}\left(x-z\right)dF_{G}^{\left(m\right)}\left(z\right)$$

for $x\in \left(0,\infty \right)$. Figure 5 illustrates the relation of $V_{t}^{\left(m\right)}$ and $W_{t}^{\left(m\right)}$.

Suppose that sensor node $s_{\tilde{m}}$ starts transmitting a packet at time t. Assume that the propagation delay from a sensor node to the sink node is negligible. Then, the packet sent by sensor node $s_{\tilde{m}}$ arrives at the sink node at time t. The packet of sensor node $s_{\tilde{m}}$ will not collide with a packet of sensor node $s_{m}$ if and only if sensor node $s_{m}$ starts transmitting no packet in the vulnerable period ${(}_{}^{}t-T^{\left(\tilde{m}\right)},t+T^{\left(\tilde{m}\right)}]$. Please note that the necessary and sufficient condition for avoiding a collision with a packet of sensor node $s_{m}$ is equivalent to the condition that

(20) $$W_{t}^{\left(m\right)}>T^{\left(\tilde{m}\right)}$$

where $W_{t}^{\left(m\right)}$ is defined in (10). Set

(21) $$\phi^{\left(m\right)}≜\underset{t\to \infty }{\lim}P{(}_{}^{}W_{t}^{\left(m\right)}>T^{\left(\tilde{m}\right)})$$

for $m\in \left\{1,\cdots ,M\right\}\setminus \left\{\tilde{m}\right\}$. Then, $\phi^{\left(m\right)}$ represents the probability that a packet of sensor node $s_{\tilde{m}}$ does not collide with a packet of sensor node $s_{m}$ at steady state. By use of (18), an exact expression of the pairwise non-collision probability $\phi^{\left(m\right)}$ is derived as follows:

(22) $$\phi^{\left(m\right)}=\frac{1}{E\left(S^{\left(m\right)}\right)}\overset{\infty }{\underset{0}{\int }}\overset{\infty }{\underset{0}{\int }}1-F_{H}^{\left(m\right)}*F_{B}^{\left(m\right)}\left(t+x\right)dtdF_{T}^{\left(\tilde{m}\right)}\left(x\right).$$

Let $\psi_{\tilde{m}}$ denote the probability that a packet of sensor node $s_{\tilde{m}}$ never collides with any other packet at steady state. Then, from (22), the non-collision probability $\psi_{\tilde{m}}$ can be expressed by

(23) $$\psi_{\tilde{m}}=\underset{m\in \left\{1,\cdots ,M\right\}\setminus \left\{\tilde{m}\right\}}{\prod }\phi^{\left(m\right)}$$

for $\tilde{m}\in \left\{1,\cdots ,M\right\}$. Let $\eta_{\tilde{m}}$ denote the nodal throughput that sensor node $s_{\tilde{m}}$ can attain. Since sensor node $s_{\tilde{m}}$ transmits a packet only once in a frame and succeeds in delivering the packet to the sink node with probability $\psi_{\tilde{m}}$, we have

(24) $$\eta_{\tilde{m}}=\frac{\psi_{\tilde{m}}}{E\left(S^{\left(\tilde{m}\right)}\right)}$$

for $\tilde{m}\in \left\{1,\cdots ,M\right\}$. From (7), (22), (23), and (24), we have an exact expression of the nodal throughput that a sensor node can achieve.

### 3.2. Exemplary Expression of Throughput

For example, suppose that harvest times $H^{\left(1\right)},\cdots ,H^{\left(M\right)}$ have an exponential distribution with mean α identically. Let H denote a random variable such that $H=H^{\left(m\right)}$ in distribution for all $m\in \left\{1,\cdots ,M\right\}$ and $F_{H}$ be the distribution function of H. Then,

(25) $$F_{H}\left(x\right)=\left\{\begin{array}{ccc} 1-e^{-\frac{x}{\alpha }} & \mathrm{if} & x>0\;\\ 0 & \mathrm{if} & x\leq 0. \end{array}\right.$$

Also, assume that back-off times $B^{\left(1\right)},\cdots ,B^{\left(M\right)}$ are identically governed by an exponential distribution with mean β. Let B denote a random variable such that $B=B^{\left(m\right)}$ in distribution for all $m\in \left\{1,\cdots ,M\right\}$. Then, B has the following distribution function.

(26) $$F_{B}\left(x\right)=\left\{\begin{array}{ccc} 1-e^{-\frac{x}{\beta }} & \mathrm{if} & x>0\;\\ 0 & \mathrm{if} & x\leq 0. \end{array}\right.$$

In addition, assume that transmission times $T^{\left(1\right)},\cdots ,T^{\left(M\right)}$ are identically distributed with an exponential distribution with mean γ. Let T denote a random variable such that $T=T^{\left(m\right)}$ in distribution for all $m\in \left\{1,\cdots ,M\right\}$. Then, T has the following distribution function.

(27) $$F_{T}\left(x\right)=\left\{\begin{array}{ccc} 1-e^{-\frac{x}{\gamma }} & \mathrm{if} & x>0\;\\ 0 & \mathrm{if} & x\leq 0. \end{array}\right.$$

Under the assumption that harvest time, back-off times and transmission times are exponentially distributed, the probability $\theta^{\left(m\right)}\left(t\right)$ in (16) is calculated by

(28) $$\theta^{\left(m\right)}\left(t\right)=P{(}_{}^{}H+B>t+y)=\frac{\alpha }{\alpha -\beta }e^{-\frac{t+y}{\alpha }}-\frac{\beta }{\alpha -\beta }e^{-\frac{t+y}{\beta }}$$

for all $m\in \left\{1,\cdots ,M\right\}$. Please note that $E{(}_{}^{}S^{\left(m\right)})=\alpha +\beta +\gamma$ since $S^{\left(m\right)}=H+B+T$ in distribution for all $m\in \left\{1,\cdots ,M\right\}$. Thus, we have

(29) $$P{(}_{}^{}W_{t}^{\left(m\right)}>y)\to \frac{\alpha^{2}}{\left(\alpha -\beta \right)\left(\alpha +\beta +\gamma \right)}e^{-\frac{y}{\alpha }}-\frac{\beta^{2}}{\left(\alpha -\beta \right)\left(\alpha +\beta +\gamma \right)}e^{-\frac{y}{\beta }}$$

as t→∞ for all $m\in \left\{1,\cdots ,M\right\}$. For sensor node $s_{\tilde{m}}$, the pairwise non-collision probability $\phi^{\left(m\right)}$ is derived by

(30) $$\phi^{\left(m\right)}=\frac{\alpha \beta \left(\alpha +\beta \right)+\gamma \left(\alpha^{2}+\alpha \beta +\beta^{2}\right)}{\left(\alpha +\gamma \right)\left(\beta +\gamma \right)\left(\alpha +\beta +\gamma \right)}$$

for all $m\in \left\{1,\cdots ,M\right\}\setminus \left\{\tilde{m}\right\}$ and the non-collision probability $\psi_{\tilde{m}}$ is also expressed by

(31) $$\psi_{\tilde{m}}={\left[\frac{\alpha \beta \left(\alpha +\beta \right)+\gamma \left(\alpha^{2}+\alpha \beta +\beta^{2}\right)}{\left(\alpha +\gamma \right)\left(\beta +\gamma \right)\left(\alpha +\beta +\gamma \right)}\right]}^{M-1}.$$

Finally, the nodal throughput that sensor node $s_{\tilde{m}}$ can attain is yielded by

(32) $$\eta_{\tilde{m}}=\frac{{\left[\alpha \beta \left(\alpha +\beta \right)+\gamma \left(\alpha^{2}+\alpha \beta +\beta^{2}\right)\right]}^{M-1}}{[\left(\alpha +\gamma \right)\left(\beta +\gamma \right){]}^{M-1}[\alpha +\beta +\gamma {]}^{M}}$$

for $\tilde{m}\in \left\{1,\cdots ,M\right\}$. Let η denote the total throughput which can be achieved in the RF energy harvesting wireless sensor network. Then, we also have

(33) $$\eta =M\eta_{\tilde{m}}=\frac{M{\left[\alpha \beta \left(\alpha +\beta \right)+\gamma \left(\alpha^{2}+\alpha \beta +\beta^{2}\right)\right]}^{M-1}}{[\left(\alpha +\gamma \right)\left(\beta +\gamma \right){]}^{M-1}[\alpha +\beta +\gamma {]}^{M}}$$

since nodal throughputs are identical.

**Figure 6:**
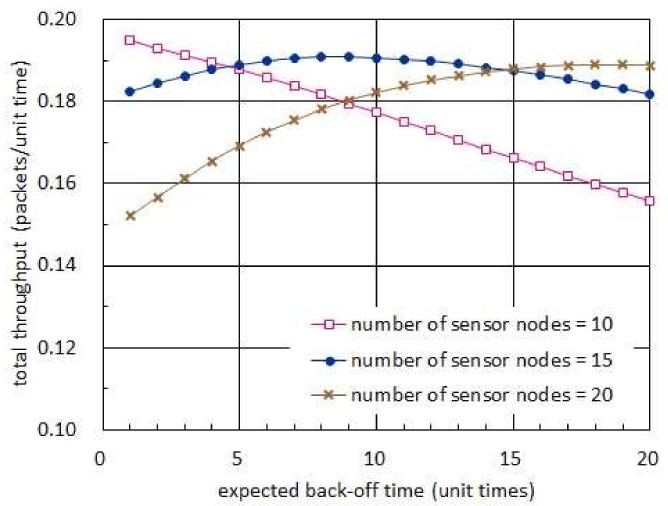
Total throughput with respect to expected back-off time.

Figure 6 shows the total throughput with respect to the expected back-off time. In this figure, the harvest times are assumed to have the exponential distribution with mean of 20 unit times while the transmission times are set to have the exponential distribution with mean of 1 unit time [16]. In addition, the back-off times are set to be exponentially distributed in an identical fashion. Figure 6 indicates that there can exist an optimal expected back-off time which maximizes the total throughput. Also, such an optimal expected back-off time is shown to increase as the number of sensor nodes increases. The existence of an optimal value is explained by the fact that the probability of packet collision decreases as the expected back-off time increases while the fraction of time that no sensor node uses for transmitting a packet increases.

## 4. Some Studies on Back-Off Time

The back-off time is a crucial factor in designing the basic MAC scheme. In this section, we consider three cases and then look for a way to improve the throughput by controlling the back-off times in each case. In the first case in which harvest times are deterministic, we find an optimal distribution for the back-off time which maximizes the total throughput. In the second case, where the harvest times are exponentially distributed, we confirm that taking proper back-off times is able to effectively enhance the total throughput. Finally, we show that shaping the distribution for the back-off time as to have high variance helps improve the total throughput.

### 4.1. Optimal Distribution for Back-Off Time

Suppose that harvest times and transmission times are deterministic. Then, once several packets collide, their collision will be definitely repeated forever unless the sensor nodes involved in the collision take back-off times. In this section, we focus on a case in which the harvest times and the transmission times are degenerated into some constants. Then, we obtain an optimal distribution for the back-off time which maximizes the total throughput.

Suppose that the harvest time $H^{\left(m\right)}$ and the transmission time $T^{\left(m\right)}$ are degenerated into positive numbers α and γ respectively, i.e.,

(34) $$\begin{array}{ccc} H^{\left(m\right)} & = & \alpha \\ T^{\left(m\right)} & = & \gamma \end{array}$$

almost surely for all $m\in \left\{1,\cdots ,M\right\}$. In addition, assume that the back-off times $B^{\left(1\right)},\cdots ,B^{\left(M\right)}$ are strictly positive random variables which are governed by a same proper distribution with mean β. Let B denote a random variable such that $B=B^{\left(m\right)}$ in distribution for all $m\in \left\{1,\cdots ,M\right\}$ and $F_{B}$ represent the distribution function of B. Then, the probability $\theta^{\left(m\right)}\left(t\right)$ in (16) is expressed by

(35) $$\theta^{\left(m\right)}\left(t\right)=1-F_{B}\left(t+y-\alpha \right)$$

for $y\in \left[0,\infty \right)$. From (17) and (35), we also have

(36) $$P{(}_{}^{}W_{t}^{\left(m\right)}>y)\to \frac{1}{\alpha +\beta +\gamma }\overset{\infty }{\underset{y-\alpha }{\int }}1-F_{B}\left(x\right)dx$$

as t→∞ for all $m\in \left\{1,\cdots ,M\right\}$. Thus, the pairwise non-collision probability $\phi^{\left(m\right)}$, i.e., the probability that a packet of sensor node $s_{\tilde{m}}$ does not collide with any packet of sensor node $s_{m}$ at steady state is yielded by

(37) $$\phi^{\left(m\right)}=\left\{\begin{array}{ccc} \frac{\alpha +\beta -\gamma }{\alpha +\beta +\gamma } & \mathrm{if} & \alpha \geq \gamma \\ \frac{1}{\alpha +\beta +\gamma }\overset{\infty }{\underset{\gamma -\alpha }{\int }}1-F_{B}\left(x\right)dx & \mathrm{if} & \alpha <\gamma \end{array}\right.$$

for all $m\in \left\{1,\cdots ,M\right\}\setminus \left\{\tilde{m}\right\}$. Since the probability $\phi^{\left(m\right)}$ is identical for all $m\in \left\{1,\cdots ,M\right\}\setminus \left\{\tilde{m}\right\}$, the non-collision probability $\psi_{\tilde{m}}$, which represents the probability that a packet of sensor node $\tilde{m}$ does not collide with any other packet at steady state, is obtained by

(38) $$\psi_{\tilde{m}}=\left\{\begin{array}{ccc} {\left(\frac{\alpha +\beta -\gamma }{\alpha +\beta +\gamma }\right)}^{M-1} & \mathrm{if} & \alpha \geq \gamma \;\\ \frac{1}{{\left(\alpha +\beta +\gamma \right)}^{M-1}}{\left[\overset{\infty }{\underset{\gamma -\alpha }{\int }}1-F_{B}\left(x\right)dx\right]}^{M-1} & \mathrm{if} & \alpha <\gamma . \end{array}\right..\;$$

Therefore, the nodal throughput that sensor node $s_{\tilde{m}}$ can achieve $s_{\tilde{m}}$ is expressed by

(39) $$\eta_{\tilde{m}}=\left\{\begin{array}{ccc} \frac{{\left(\alpha +\beta -\gamma \right)}^{M-1}}{{\left(\alpha +\beta +\gamma \right)}^{M}} & \mathrm{if} & \alpha \geq \gamma \;\\ \frac{1}{{\left(\alpha +\beta +\gamma \right)}^{M}}{\left[\overset{\infty }{\underset{\gamma -\alpha }{\int }}1-F_{B}\left(x\right)dx\right]}^{M-1} & \mathrm{if} & \alpha <\gamma . \end{array}\right.$$

Moreover, the total throughput is obtained by

(40) $$\eta =\left\{\begin{array}{ccc} \frac{M{\left(\alpha +\beta -\gamma \right)}^{M-1}}{{\left(\alpha +\beta +\gamma \right)}^{M}} & \mathrm{if} & \alpha \geq \gamma \\ \frac{M}{{\left(\alpha +\beta +\gamma \right)}^{M}}{\left[\overset{\infty }{\underset{\gamma -\alpha }{\int }}1-F_{B}\left(x\right)dx\right]}^{M-1} & \mathrm{if} & \alpha <\gamma \end{array}\right.$$

since nodal throughputs are identical.

First, consider the case that α≥γ. Please note that it is a practically feasible case since the harvest time is typically much longer than the transmission time in practice. By differentiating the total throughput η with respect to the expected back-off time β and then equating it to zero, we obtain a critical point, denoted by $\hat{\beta }$ as follows.

(41) $$\hat{\beta }=\left(2M-1\right)\gamma -\alpha .$$

Please note that $\hat{\beta }$ is also a global maximum point. However, as far as $\alpha <\left(2M-1\right)\gamma$, $\hat{\beta }$ is the optimal expected back-off time since the expected back-off time β should be strictly positive. Otherwise, there exists no optimal expected back-off time. Please note that no specific distribution for the back-off time has been assumed until the optimal expected back-off time in (31) is derived. Thus, an optimal back-off time is proved not to be characterized by the distribution but only by the expected value. Suppose that $\gamma \leq \alpha <\left(2M-1\right)\gamma$. Let $\eta^{*}$ denote the maximum total throughput. Then, we have

(42) $$\eta^{*}=\frac{1}{2\gamma }{\left(1-\frac{1}{M}\right)}^{M-1}$$

when $\gamma \leq \alpha <\left(2M-1\right)\gamma$. On the other hand,

(43) $$\eta \to \frac{M{\left(\alpha -\gamma \right)}^{M-1}}{{\left(\alpha +\gamma \right)}^{M}}$$

as β→0 when $\alpha >\left(2M-1\right)\gamma$.

Secondly, consider the case that 0<α<γ. Since

(44) $$0\leq \overset{\gamma -\alpha }{\underset{0}{\int }}1-F_{B}\left(x\right)dx\leq \gamma -\alpha ,$$

the total throughput η is bounded as follows.

(45) $$\frac{M\cdot \max{\left\{0,\alpha +\beta -\gamma \right\}}^{M-1}}{{\left(\alpha +\beta +\gamma \right)}^{M}}\leq \eta \leq \frac{M\beta^{M-1}}{{\left(\alpha +\beta +\gamma \right)}^{M}}.$$

In (45), the upper bound on the total throughput has a critical point, denoted by $\overset{ˇ}{\beta }$, as follows.

(46) $$\overset{ˇ}{\beta }=\left(M-1\right)\left(\alpha +\gamma \right).$$

Please note that $\overset{ˇ}{\beta }$ is also a global maximum point. Replacing β with $\hat{\beta }$ in the lower bound as well as β with $\overset{ˇ}{\beta }$ in the upper bound shown in (45), we obtain upper and lower bounds on the maximum total throughput, denoted by $\eta^{*}$, as follows.

(47) $$\frac{1}{2\gamma }{\left(1-\frac{1}{M}\right)}^{M-1}\leq \eta^{*}\leq \frac{1}{\alpha +\gamma }{\left(1-\frac{1}{M}\right)}^{M-1}.$$

**Figure 7:**
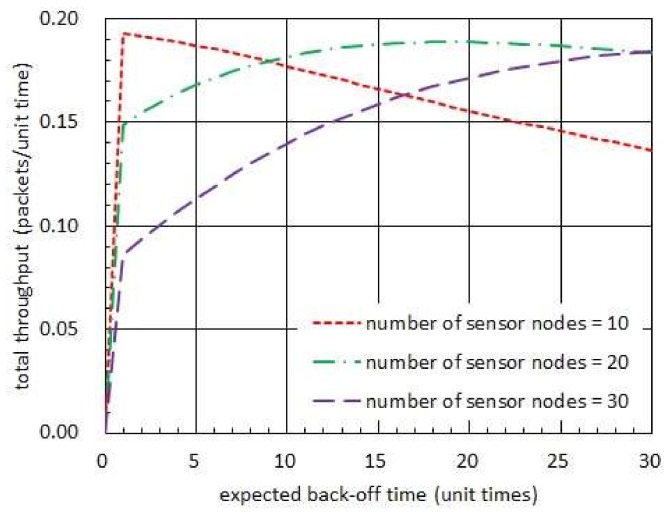
Total throughput with respect to expected back-off time when harvest time is longer than transmission time.

Figure 7 shows the total throughput with respect to the expected back-off time. In this figure, the harvest times are fixed to 20 unit times while the transmission times are set to be 1 unit time [16]. In addition, the back-off times are set to have an exponential distribution. In Figure 7, we observe that there exists an optimal expected back-off time, which maximizes the total throughput, when the number of sensor nodes is 20 or 30. On the other hand, there is no optimal value in case 10 sensor nodes reside in the network. Such a dichotomy of the expected values of back-off time corresponds to the one which results in (42) and (43), respectively. In addition, we notice that the optimal expected back-off time increases as the number of sensor nodes increases.

**Figure 8:**
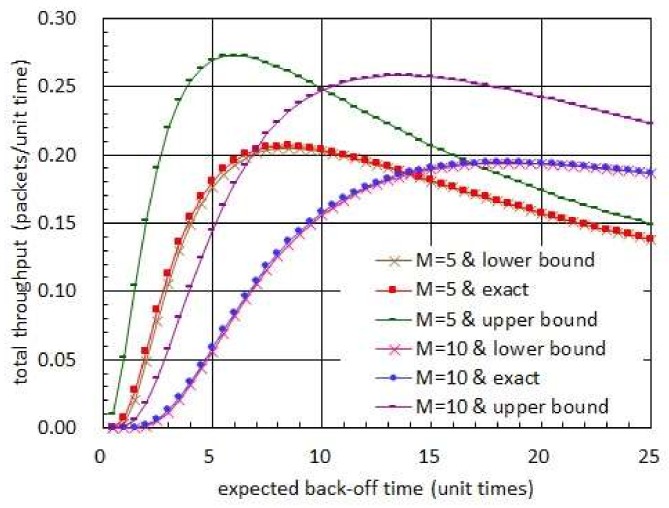
Total throughput with respect to expected back-off time when harvest time is shorter than transmission time.

Figure 8 shows the total throughput with respect to the expected back-off time. In this figure, the harvest times are assumed to be fixed to 0.5 unit times while the transmission times are set to be 1 unit time. Also, the back-off times are set to have an exponential distribution. In figure 8, we observe that the total throughput is well bounded by the upper and lower bounds given in (45). In particular, we notice that the lower bound is highly tighter than the upper bound. Moreover, the optimal expected back-off time is fairly close to the value of expected back-off time which maximizes the lower bound.

### 4.2. Effectiveness of Taking Back-Off Time

Suppose that the harvest times are not deterministic. Then, due to the random fluctuations in harvest times, a collision of packets may be never repeated even if the sensor nodes, which are involved in the collision, take no back-off time at all. Furthermore, without taking back-off times, a sensor node is able to attempt to deliver packets more frequently. In this section, we consider two cases; sensor nodes take exponentially distributed back-off times in a case and sensor nodes take no back-off time in the other case. Then, we investigate whether taking positive back-off times can help enhance the throughput or not.

First, consider the case in which sensor nodes take positive back-off times. Suppose that the harvest times $H^{\left(1\right)},\cdots ,H^{\left(M\right)}$ are governed by a same exponential distribution with mean α. Let H denote a random variable such that $H=H^{\left(m\right)}$ for all $m\in \left\{1,\cdots ,M\right\}$. The distribution function of H, denoted by $F_{H}$, is given in (17). In this case, the back-off times $B^{\left(1\right)},\cdots ,B^{\left(M\right)}$ are set to identically have an exponential distribution with mean β. Let B denote a random variable such that $B=B^{\left(m\right)}$ in distribution for all $m\in \left\{1,\cdots ,M\right\}$. The distribution function of B, denoted by $F_{B}$, is given in (18). In addition, the transmission times $T^{\left(1\right)},\cdots ,T^{\left(M\right)}$ are set to be degenerated into a positive number γ, i.e., $T^{\left(m\right)}=\gamma$ almost surely for all $m\in \left\{1,\cdots ,M\right\}$.

In the case that sensor nodes take positive back-off times, the probability $\theta^{\left(m\right)}\left(t\right)$ in (16) is calculated by

(48) $$\theta^{\left(m\right)}\left(t\right)\;=\frac{\alpha }{\alpha -\beta }e^{-\frac{1}{\alpha }\left(t+y\right)}-\frac{\beta }{\alpha -\beta }e^{-\frac{1}{\beta }\left(t+y\right)}$$

for $y\in \left[0,\infty \right)$. Thus, for sensor node $s_{\tilde{m}}$, the pairwise non-collision probability $\phi^{\left(m\right)}$ in (22) is yielded by

(49) $$\phi^{\left(m\right)}=\frac{1}{\alpha +\beta +\gamma }{[}_{}^{}\frac{\alpha^{2}}{\alpha -\beta }e^{-\frac{\gamma }{\alpha }}-\frac{\beta^{2}}{\alpha -\beta }e^{-\frac{\gamma }{\beta }}]$$

for all $m\in \left\{1,\cdots ,M\right\}\setminus \left\{\tilde{m}\right\}$ and the non-collision probability $\psi_{\tilde{m}}$ in (23) is also yielded by

(50) $$\psi_{\tilde{m}}\;=\frac{1}{{\left[\alpha +\beta +\gamma \right]}^{M-1}}{\left[\frac{\alpha^{2}}{\alpha -\beta }e^{-\frac{\gamma }{\alpha }}-\frac{\beta^{2}}{\alpha -\beta }e^{-\frac{\gamma }{\beta }}\right]}^{M-1}$$

for all $\tilde{m}\in \left\{1,\cdots ,M\right\}$. Therefore, the nodal throughput that sensor node $s_{\tilde{m}}$ can attain is obtained by

(51) $$\eta_{\tilde{m}}=\frac{1}{{\left(\alpha +\beta +\gamma \right)}^{M}}{\left[\frac{\alpha^{2}}{\alpha -\beta }e^{-\frac{\gamma }{\alpha }}-\frac{\beta^{2}}{\alpha -\beta }e^{-\frac{\gamma }{\beta }}\right]}^{M-1}$$

for all $\tilde{m}\in \left\{1,\cdots ,M\right\}$ and the total throughput is also expressed by

(52) $$\eta =\frac{M}{{\left(\alpha +\beta +\gamma \right)}^{M}}{\left[\frac{\alpha^{2}}{\alpha -\beta }e^{-\frac{\gamma }{\alpha }}-\frac{\beta^{2}}{\alpha -\beta }e^{-\frac{\gamma }{\beta }}\right]}^{M-1}.$$

Secondly, consider the case in which sensor nodes take no back-off times. Suppose that the harvest times $H^{\left(1\right)},\cdots ,H^{\left(M\right)}$ are governed by a same exponential distribution with mean α. In this case, the back-off times $B^{\left(1\right)},\cdots ,B^{\left(M\right)}$ are set to be degenerated into 0, i.e., $B^{\left(m\right)}=0$ almost surely for all $m\in \left\{1,\cdots ,M\right\}$. Also, the transmission times $T^{\left(1\right)},\cdots ,T^{\left(M\right)}$ are set to be degenerated into a positive number γ.

In the case that sensor nodes take no back-off time, the probability $\theta^{\left(m\right)}\left(t\right)$ given in (16) is calculated by

(53) $$\theta^{\left(m\right)}\left(t\right)\;=e^{-\frac{1}{\alpha }\left(t+y\right)}$$

for $y\in \left[0,\infty \right)$. Thus, for sensor node $s_{\tilde{m}}$, the pairwise non-collision probability $\phi^{\left(m\right)}$ in (22) is yielded by

(54) $$\phi^{\left(m\right)}=\frac{\alpha }{\alpha +\gamma }e^{-\frac{\gamma }{\alpha }}$$

for all $m\in \left\{1,\cdots ,M\right\}\setminus \left\{\tilde{m}\right\}$ and the non-collision probability $\psi_{\tilde{m}}$ in (23) is also yielded by

(55) $$\psi_{\tilde{m}}={\left(\frac{\alpha }{\alpha +\gamma }\right)}^{M-1}e^{-\left(M-1\right)\frac{\gamma }{\alpha }}$$

for all $\tilde{m}\in \left\{1,\cdots ,M\right\}$. Therefore, the nodal throughput that sensor node $s_{\tilde{m}}$ can attain is obtained by

(56) $$\eta_{\tilde{m}}=\frac{\alpha^{M-1}}{{\left(\alpha +\gamma \right)}^{M}}e^{-\left(M-1\right)\frac{\gamma }{\alpha }}$$

for all $\tilde{m}\in \left\{1,\cdots ,M\right\}$ and the total throughput is also obtained by

(57) $$\eta =\frac{M\alpha^{M-1}}{{\left(\alpha +\gamma \right)}^{M}}e^{-\left(M-1\right)\frac{\gamma }{\alpha }}.$$

**Figure 9:**
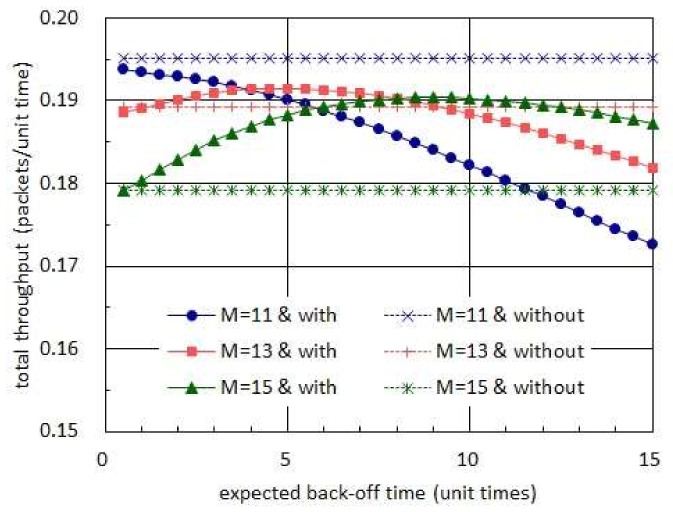
Comparison of total throughputs achieved with and without taking back-off times.

Figure 9 compares the total throughputs which are, respectively, achieved with and without taking back-off times. In this figure, the harvest times are assumed to have the exponential distribution with mean of 20 unit times while the transmission times are set to be almost surely equal to 1 unit time [16]. In addition, the back-off times are set to be governed by an exponential distribution in case sensor nodes take positive back-off times. In this figure, we observe that taking no back-off time is always able to achieve higher total throughput than taking exponentially distributed back-off times when the number of sensor nodes is 11. However, in the network consisting of 13 or 15 sensor nodes, we notice that taking proper back-off times can effectively improve the total throughput.

### 4.3. Shaping Distribution for Back-Off Time

Apparently, the shape of the distribution for the back-off time affects the throughput that the basic MAC scheme is able to attain. Please note that the variance of a distribution is a key parameter which determines the shape of the distribution. In this section, we assume a situation in which the sum of harvest and back-off times is governed by an Erlang distribution. Then, we examine the dominant tendency that the total throughput shows as the variance of back-off time changes.

First, suppose that the harvest times $H^{\left(1\right)},\cdots ,H^{\left(M\right)}$ are identically distributed with mean α. Let H denote a random variable such that $H=H^{\left(m\right)}$ in distribution for all $m\in \left\{1,\cdots ,M\right\}$. Also, let $F_{H}$ denote the distribution function of H and $\Phi_{H}$ be the Laplace-Stieltjes transform of $F_{H}$, i.e.,

(58) $$\Phi_{H}\left(s\right)=\overset{\infty }{\underset{0}{\int }}e^{-sx}dF_{H}\left(x\right)$$

for complex number s. Secondly, suppose that the back-off times $B^{\left(1\right)},\cdots ,B^{\left(M\right)}$ are governed by a same distribution with mean β. Let B denote a random variable such that $B=B^{\left(m\right)}$ in distribution for all $m\in \left\{1,\cdots ,M\right\}$ and $F_{B}$ be the distribution function of H. Then, the random variable H+B is set to have the Erlang distribution with shape parameter ν and rate parameter λ, i.e.,

(59) $$F_{H}*F_{B}\left(x\right)=1-\overset{\nu -1}{\underset{k=0}{\sum }}\frac{e^{-\lambda x}{\left(\lambda x\right)}^{k}}{k!}$$

for $x\in \left(0,\infty \right)$. The Laplace-Stieltjes transform of $F_{H}*F_{B}$ is calculated by

(60) $$\Phi_{H+B}\left(s\right)={\left[\frac{\lambda }{s+\lambda }\right]}^{\nu }.$$

Please note that $F_{B}$ is equal to the inverse Laplace-Stieltjes transform of $\Phi_{H+B}\left(s\right)/\Phi_{H}\left(s\right)$. Thirdly, suppose that the transmission times $T^{\left(1\right)},\cdots ,T^{\left(M\right)}$ are degenerated into a positive number γ, i.e.,

(61) $$T^{\left(m\right)}=\gamma$$

almost surely for all $m\in \left\{1,\cdots ,M\right\}$.

In case the sum of harvest and back-off times has an Erlang distribution, the probability $\theta^{\left(m\right)}\left(t\right)$ in (16) is obtained by

(62) $$\theta^{\left(m\right)}\left(t\right)=\overset{\nu -1}{\underset{k=0}{\sum }}\frac{e^{-\lambda \left(t+y\right)}{\left[\lambda \left(t+y\right)\right]}^{k}}{k!}$$

for $y\in \left[0,\infty \right)$. Then,

(63) $$P(W_{t}^{\left(m\right)}>y)\to \frac{1}{\left(\alpha +\beta +\gamma \right)\lambda }\overset{\nu -1}{\underset{k=0}{\sum }}\overset{k}{\underset{n=0}{\sum }}\frac{e^{-\lambda y}{\left(\lambda y\right)}^{k}}{n!}$$

as t→∞. From (63), the pairwise non-collision probability $\phi^{\left(m\right)}$ is yielded by

(64) $$\phi^{\left(m\right)}=\frac{1}{\left(\alpha +\beta +\gamma \right)\lambda }\overset{\nu -1}{\underset{k=0}{\sum }}\overset{k}{\underset{n=0}{\sum }}\frac{e^{-\lambda \gamma }{\left(\lambda \gamma \right)}^{n}}{n!}$$

for all $m\in \left\{1,\cdots ,M\right\}\setminus \left\{\tilde{m}\right\}$ and the non-collision probability $\psi_{\tilde{m}}$ is also yielded by

(65) $$\psi_{\tilde{m}}=\frac{1}{{\left(\alpha +\beta +\gamma \right)}^{M-1}\lambda^{M-1}}{\left[\overset{\nu -1}{\underset{k=0}{\sum }}\overset{k}{\underset{n=0}{\sum }}\frac{e^{-\lambda \gamma }{\left(\lambda \gamma \right)}^{n}}{n!}\right]}^{M-1}$$

for all $\tilde{m}\in \left\{1,\cdots ,M\right\}$. From (65), the nodal throughput $\eta_{\tilde{m}}$ is expressed by

(66) $$\eta_{\tilde{m}}=\frac{1}{{\left(\alpha +\beta +\gamma \right)}^{M}\lambda^{M-1}}{\left[\overset{\nu -1}{\underset{k=0}{\sum }}\overset{k}{\underset{n=0}{\sum }}\frac{e^{-\lambda \gamma }{\left(\lambda \gamma \right)}^{n}}{n!}\right]}^{M-1}$$

for all $\tilde{m}\in \left\{1,\cdots ,M\right\}$ and the total throughput η is also obtained by

(67) $$\eta =\frac{M}{{\left(\alpha +\beta +\gamma \right)}^{M}\lambda^{M-1}}{\left[\overset{\nu -1}{\underset{k=0}{\sum }}\overset{k}{\underset{n=0}{\sum }}\frac{e^{-\lambda \gamma }{\left(\lambda \gamma \right)}^{n}}{n!}\right]}^{M-1}.$$

**Figure 10:**
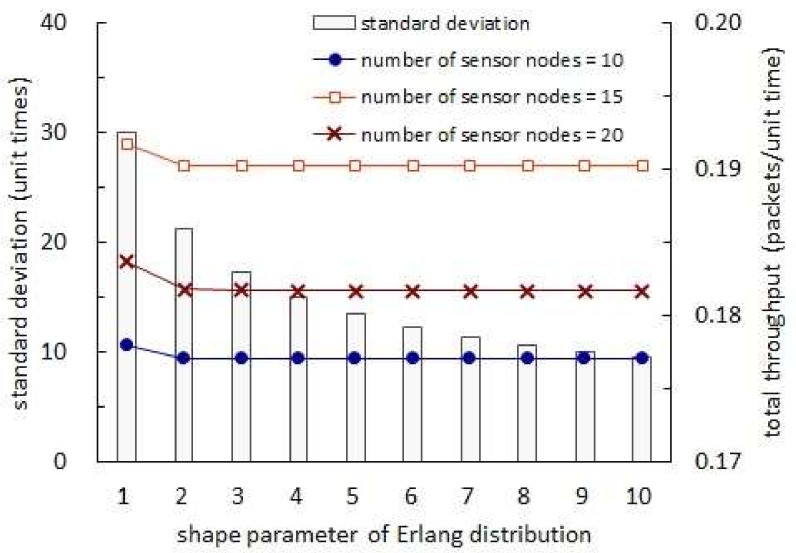
Total throughput and standard deviation with respect to shape parameter of Erlang distribution.

Figure 10 shows the total throughput with respect to the shape parameter of Erlang distribution. In this figure, a specific distribution is not assumed for the harvest times. Given distribution for the harvest times, however, the back-off times are set to have a certain distribution so that the sum of harvest and back-off times are governed by an Erlang distribution with mean of 30 unit times. In addition, the transmission times are set to be 1 unit time almost surely. In Figure 10, we observe that the total throughput is reduced as the shape parameter is increased. First, note that the mean of the sum of harvest and back-off times is set to be unchanged with respect to the shape parameter. Thus, the mean of the back-off time is fixed in this figure. Secondly, note that the mean and variance of the Erlang distribution with shape parameter ν and rate parameter λ are equal to ν/λ and $\nu /\lambda^{2}$, respectively. Thus, as the shape parameter increases, the variance of the sum of harvest and back-off times decreases and hence the variance of the back-off time decreases. (Figure 10 illustrates the standard deviation which decreases as the shape parameter increases.) The two arguments above corroborate the conclusion that shaping the back-off time so that its variance is increased while its mean is not changed contributes to the throughput enhancement. Please note that all of the expected harvest time, expected back-off time and expected transmission time are fixed regardless of the shape parameter in Figure 10. Thus, the phenomenon stated in the conclusion above takes place since the probability of packet collision decreases as the shape parameter increases while the fraction of time that no sensor node uses for transmitting a packet is unchanged.

## 5. Conclusions

RF energy harvesting technologies provided us with a new paradigm for wireless sensor networks; a wireless sensor network is able to live an eternal life without replacement or recharge of batteries. Against theoretical expectations; however, an RF energy harvesting wireless sensor network suffers from a scarcity of energy in practice and, consequently, naïveté has to be within a MAC scheme that supports sensor nodes to deliver their packets to a sink node. Upon perception of the difficulties in RF energy harvesting wireless sensor networks, our practical choice was a basic contending-type MAC scheme rooted in ALOHA. In the basic MAC scheme, each sensor node simply repeats harvesting energy, backing off for a while and transmitting a packet. Since the basic MAC scheme belongs to ALOHA clan; however, it may bring about a collision among some packets. In turn, such a collision leads to the loss of involving packets and hence deteriorates the throughput. Using the renewal theory, we thus derived an exact expression of the throughput that the basic MAC scheme is able to attain. Then, by use of the throughput formula, we sought strategies of controlling back-off times as to improve the throughput. In case studies, we first revealed that an optimal back-off time, which maximizes the total throughput, is not characterized by the distribution but only by the mean value when the harvest times are deterministic. Secondly, we confirmed that taking proper back-off times is able to effectively improve the throughput even when the harvest times are random. Thirdly, we showed that shaping the back-off time so that its variance is increased while its mean is not changed can help ameliorate the throughput that the basic MAC scheme is able to achieve.

## Figures and Tables

**Figure 5:**
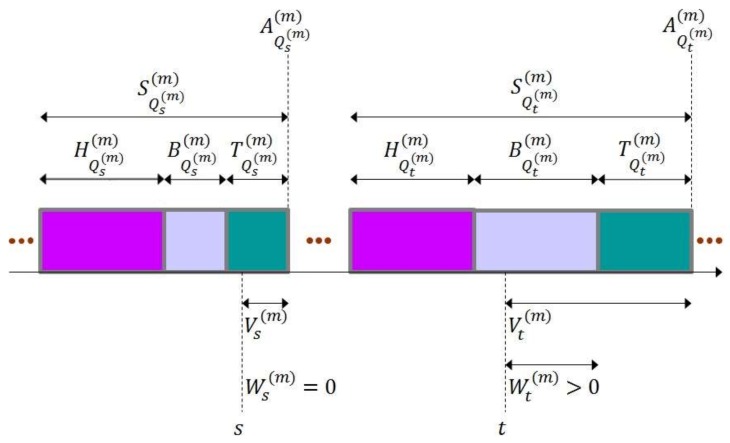
Relation of $V_{t}^{\left(m\right)}$ and $W_{t}^{\left(m\right)}$.
